# Needs and availability of medical specialists’ and allied health professionals’ visits in German nursing homes: a cross-sectional study of nursing home staff

**DOI:** 10.1186/s12913-020-05169-7

**Published:** 2020-04-21

**Authors:** Ann-Kristin Schröder, Alexander Maximilian Fassmer, Katharina Allers, Falk Hoffmann

**Affiliations:** Department of Health Services Research, School VI - Medicine and Health Sciences, Carl von Ossietzky University of Oldenburg, Oldenburg, Germany

**Keywords:** Nursing homes, Medical specialists, Allied health professionals, Needs, Survey

## Abstract

**Background:**

The medical care for nursing home residents is estimated to be partly inadequate in Germany. The aim of this study is to investigate the needs and utilization of general practitioners (GPs), medical specialists and allied health professionals.

**Methods:**

A survey was sent to a nationwide random sample of 1069 nursing homes in Germany in January 2019. Nursing staff managers were asked about medical care. Regular nursing home visits by medical specialists and allied health professionals were defined as at least one contact per year to at least one nursing home resident.

**Results:**

A total of 486 persons responded (45.5%). On average, nursing homes have contact to 8.6 (interquartile range: 4–10) different GPs. Almost 70% of respondents agreed that residents’ medical care should be coordinated by GPs. However, only 46.0% stated that specialist treatment should require GP referral. A high need was seen for care from physiotherapists (91.0%), neurologists or psychiatrists (89.3%), dentists (73.7%), and urologists (71.3%). Regarding the actual utilization of medical specialists and health professionals, most nursing homes have regular contact to physiotherapists (97.1%), psychiatrists or neurologists (90.4%), speech therapists (85.0%), and dentists (84.8%). Remarkable discrepancies between need and utilization were found for urologists and ophthalmologists.

**Conclusion:**

There is large variance in the number of GPs per nursing home, and needs for medical specialists, especially urologists and ophthalmologists, seem unmet. Interprofessional collaboration between GPs, medical specialists and allied health professionals should be improved, and GPs should play a more coordinating role.

## Background

Due to demographic changes, the growing number of people in need of care has become a worldwide challenge [1]. In Germany, for instance, approximately one quarter of the 3.4 million people in need of care are living in nursing homes [2]. Nursing home residents are characterized by a high prevalence of different health problems including dementia [3, 4], urinary and faecal incontinence [5, 6], decubitus [7, 8], poor dental health [9, 10], malnutrition [11, 12], frailty [13] as well as visual [14, 15] and hearing impairment [16, 17]. These health problems affect nursing home residents’ quality of life and might lead to a high need for medical care [18].

In Germany, general practitioners (GPs), medical specialists and allied health professionals are not regularly employed by nursing homes. Like all German patients, nursing home residents have the right to freely choose their physicians and allied health professionals [19]. However, access to medical care is often limited due to reduced mobility and multimorbidity, and over 81% of nursing home residents are incapable of visiting physicians outside their nursing home [20]. Consequently, GPs, medical specialists and allied health professionals have to visit the nursing home [4]. GPs have the most contacts to nursing home residents, but often, many different GPs care for patients in a single nursing home [21, 22]. Whereas neurologists and psychiatrists have regular contacts to nursing home residents, contact frequencies to other medical specialists, especially to ophthalmologists, gynaecologists, otorhinolaryngologists and dentists are lower [9, 11, 14, 20].

Previous studies have concluded that the medical care for nursing home residents is often inadequate in Germany, but most studies included data of few nursing homes or only administrative data [23, 20]. Comprehensive and representative data about medical care needs of nursing home residents, the number of visiting medical specialists and contact frequencies in Germany are lacking. Also, little is known about visits of different allied health professionals like physiotherapists, speech therapists or optometrists in nursing homes [4, 24].

Therefore, the aim of this study is to investigate the estimated needs and consultation of GPs, various medical specialists and allied health professionals in nursing homes in Germany.

## Methods

### Study design and target population

This cross-sectional study was part of the publicly funded HOMERN project (HOspitalisations and eMERgency department visits of Nursing home residents) [25] which aims to improve medical care for nursing home residents.

For this survey we randomly selected 1121 nursing homes from the approximately 11,200 facilities providing long-term care in Germany, searchable by the Care Navigator offered by the Federal Association of Local Health Insurance Funds (“AOK Pflege-Navigator”). After checking the sample manually, we excluded 52 facilities caring for children, patients with prolonged mechanical ventilation or in persistent vegetative state. Each of the remaining 1069 NHs received a questionnaire in January 2019 that was preferably addressed to the nursing staff manager. For that, we searched their names manually. If the present nursing staff manager was not detectable, we searched for the facility director or the executive board instead. Only if we could not find any contact person, we addressed the questionnaire to the current nursing staff manager without naming a person.

A reminder was sent to all nursing homes two weeks later. Several strategies shown by a Cochrane review to increase response to postal surveys, including use of a short questionnaire, follow-up contact, inclusion of a copy of the questionnaire at follow-up, personalized letters, and academic origin of the study, were applied [26]. Participation in this study was entirely voluntary; data were collected anonymously.

The study received a waiver by the local medical ethics committee of the Carl von Ossietzky University of Oldenburg (no. 2018–147).

### Questionnaire

An interdisciplinary team developed the four-page questionnaire containing questions about medical care provision in nursing homes, emergency department and hospital use as well end-of-life care. The questionnaire has been published previously [27].

This present study solely reports on medical care in nursing homes. Different aspects of the medical care provided to nursing home residents were requested. In the first part, participants were asked general questions on medical care provided by GPs. With the questions “Do you agree that medical care for nursing home residents should be coordinated by the GP?” and “Do you think that medical treatment for nursing home residents should only be provided after referral by the GP?” we wanted to investigate how nursing home staff estimates the role of the GPs. Furthermore, participants were asked to what extent they agree with different statements about medical care provision by GPs, their practice teams, telemedicine and the out-of-hours medical care by the Associations of Statutory Health Insurance Physicians (Ärztlicher Bereitschaftsdienst) on a 5-point Likert scale (0= ‘I strongly disagree’ to 4= ‘I strongly agree’). This service by the Associations of Statutory Health Insurance Physicians is intended for patients who require outpatient medical treatment in urgent medical cases and who are not in a life-threatening situation [28].

In addition, we asked the question “How do you estimate the need of medical specialists/allied health professionals in nursing homes?” to estimate the actual need for different medical specialists (ophthalmologists, surgeons/orthopaedists, dermatologists, gynaecologists, otorhinolaryngologists, psychiatrists/neurologists, urologists and dentists) and allied health professionals (physiotherapists, speech therapists, optometrists, hearing aid acousticians, nutritionists and social workers) using a 5-point Likert scale (0 = ‘very low’ to 4 = ‘very high’). In addition, the question “Which of the following listed professional groups conduct regular visits in your nursing home (i.e. at least one visit per year for at least one nursing home resident)?” was asked in order to assess the actual utilization of medical specialists and allied health professionals. Participants also could indicate the number of GPs, medical specialists and allied health professionals conducting regular visits in their nursing home.

Besides, the following sociodemographic characteristics of participants were requested: age, sex, current position, and number of years working in this position. Also, nursing home characteristics were surveyed, including the number of beds, type of sponsorship (private, non-profit, local), region, location of the nursing home (rural ≤20,000; semi-urban between > 20,000 and ≤ 100,000; urban > 100,000 inhabitants), and distance to the nearest hospital with an emergency department.

### Statistical analysis

Analyses were performed using descriptive statistics. Continuous data are presented as means, standard deviation (SD), median and interquartile range (IQR). For categorical data, frequencies were calculated. For the rating of personal attitudes on the 5-point Likert scale, we present summarized proportions for the categories ‘strongly agree’ and ‘agree’ as well as ‘very high’ and ‘high’. Since not all participants answered every question in the questionnaire, the analyses were restricted to subjects with no missing values given in the respective questions. Statistical analysis was performed with SPSS 25.0 (SPSS Inc., Chicago, IL, USA) and SAS 9.4 (SAS Institute Inc., Cary, NC, USA).

## Results

### Baseline data on respondents and nursing homes

A total of 486 nursing homes answered the questionnaire, which is a response of 45.5%. The mean age of respondents was 48.0 years (SD: 9.8; IQR: 40–56), and most were female (71.0%). Most respondents were nursing staff managers (64.7%). In total, participants had a mean work experience of 9.7 years (SD: 8.0; IQR: 3–15).

Most of the nursing homes were non-profit facilities (52.7%). On average, they had 89.1 beds, and most provided between 51 and 100 beds (Table 1). More than a half of the nursing homes (51.6%) were located in rural areas. The mean distance to the nearest hospital with an emergency department was 8.5 km (SD: 7.8; IQR: 3–12). Most nursing homes were located in the Western federal states of Germany (35.9%), the least in the East (15.1%).

**Table 1:** Characteristics of responding nursing homes

Characteristic	Proportion
**Type of sponsorship (*n*** **= 469)**^**a**^	
private	39.2%
non-profit	52.7%
local	8.1%
**Number of beds (*n*** **= 478)**^**a**^ mean (SD) [IQR]	89.1 (47.5) [60–108]
≤ 50	18.6%
51–100	51.0%
101–150	23.4%
151–200	5.0%
> 200	1.9%
**Location of the nursing home (*n*** **= 461)**^**a**^	
Rural ≤20,000	51.6%
Semiurban > 20,000- ≤100,000	28.4%
Urban > 100,000	20%
**Distance to nearest hospital with emergency department (*n*** **= 419)**^**a**^**in kilometres, mean (SD) [IQR]**	8.5 (7.8) [3–12]
< 1	4.8%
1–5	45.6%
6–10	21.2%
11–15	14.3%
16–20	7.6%
21–25	3.1%
> 25	3.3%
**Area (*n*** **= 465)**	
North Germany ^1^	22.4%
East Germany ^2^	15.1%
West Germany^3^	35.9%
South Germany^4^	26.7%

^a^Numbers differ because of missing values

*SD* standard deviation, *IQR* interquartile range.

^1^Includes Lower Saxony, Bremen, Hamburg, Schleswig-Holstein, Mecklenburg-Western Pomerania

^2^Includes Berlin, Brandenburg, Saxony, Saxony-Anhalt, Thuringia

^3^Includes North Rhine-Westphalia, Rhineland Palatinate, Saarland, Hesse

^4^Includes Bavaria, Baden-Wuerttemberg

### Contact to GPs and medical care for nursing home residents

**Fig. 1:**
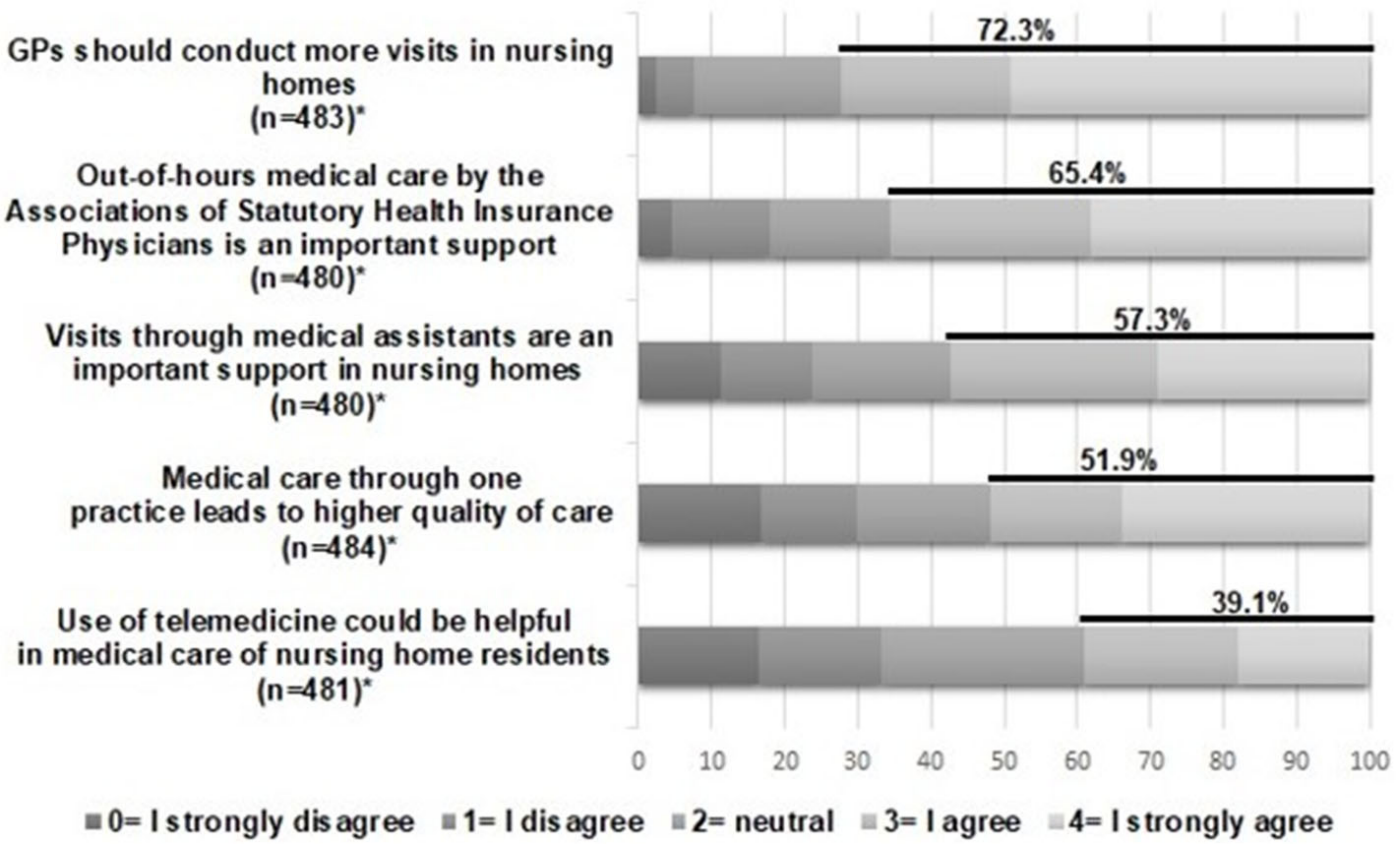
Perceptions on medical care for nursing home residents Detailed legend: *Numbers differ because of missing values

In the average nursing home, 8.6 (SD: 7.6; IQR: 4–10) different GPs provided care to at least one resident. A total of 69.9% of respondents agreed that the medical care for nursing home residents should be coordinated by GPs. About half of respondents agreed that specialist medical treatment of residents should require GP referral (46.0%) and that all residents should be treated by just one instead of different GP practices (51.9%). Nearly two-thirds (65.4%) of respondents agreed that out-of-hours medical care is important to support the medical care for nursing home residents and 72.3% agreed with the statement that GPs should conduct visits in nursing homes more often (Fig. 1; for detailed data see Additional file 1). Only 39.1% agreed that telemedical advice is helpful for medical care provision in nursing homes.

### Estimated need for and actual utilization of medical specialists

**Fig. 2:**
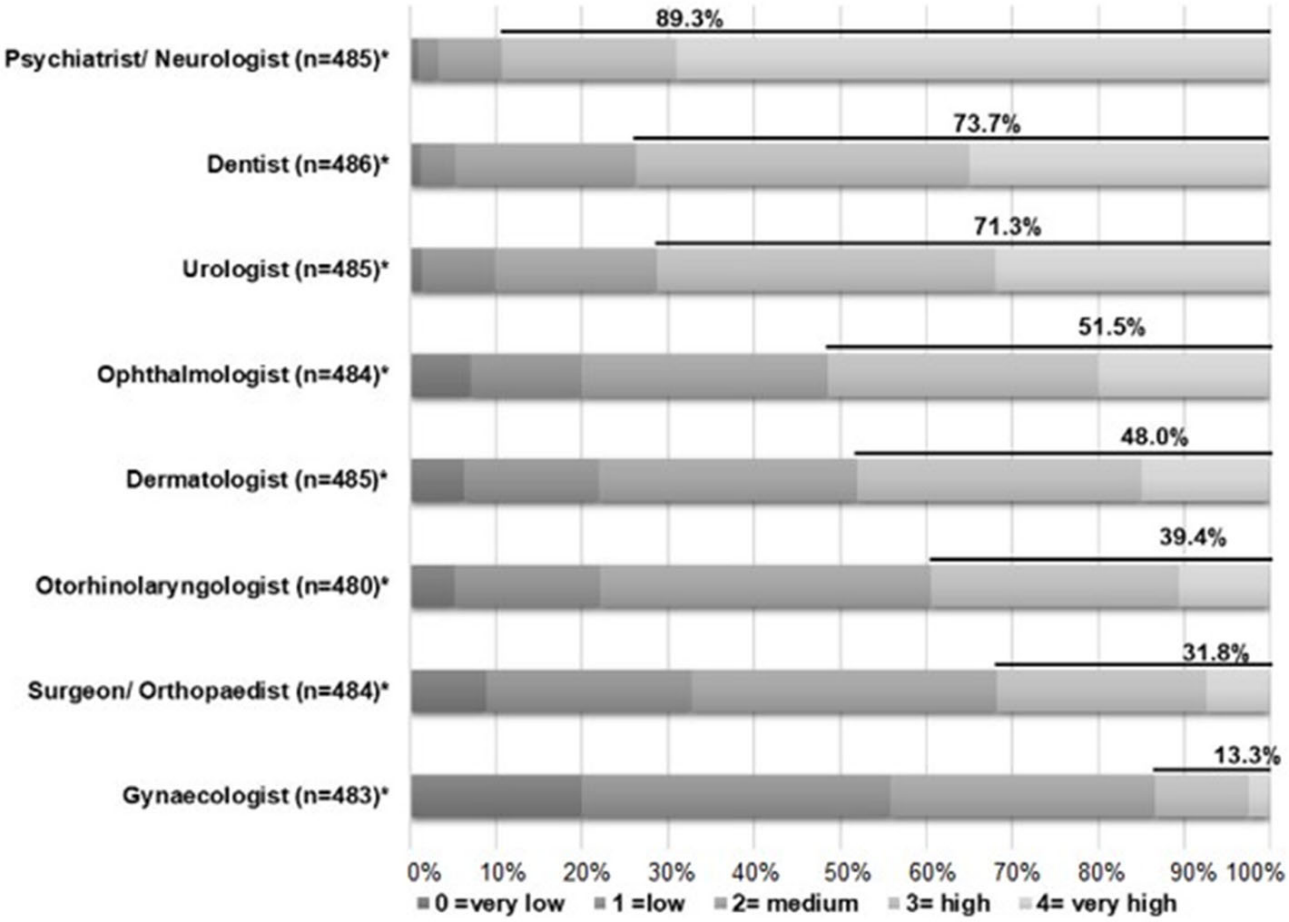
Estimated need for various medical specialists Detailed legend: *Numbers differ because of missing values

Regarding different medical specialists, 89.3% of the respondents estimated a high need in the care provision by psychiatrists or neurologists (Fig. 2; for detailed data see Additional file 2). Furthermore, 73.7, 71.3, 51.5 and 48.0% of the respondents estimated the need for dentists, urologists, ophthalmologists and dermatologists as high. Smaller proportions saw a high need for gynaecologists, surgeons or orthopaedists and otorhinolaryngologists.

Regarding the actual utilization of medical specialists, 90.4% of the nursing homes indicated regular contact of residents to a psychiatrist or neurologist in the last 12 months (Table 2). Contact to dentists was reported by 84.8% of nursing homes, and contact to urologists, by 57.3%. The proportion was substantially lower for regular contact to an otorhinolaryngologist (38.8%) and for contact to an ophthalmologist (18.1%). The group of psychiatrists and neurologists was represented by the highest number of specialists per nursing home, at a mean of 1.6 psychotherapists or neurologists (SD: 0.8; IQR: 1–2) per nursing home (Table 2). The average nursing home was attended by 1.2 otorhinolaryngologists and ophthalmologists.

**Table 2:** Contacts of nursing homes to various medical specialists and allied health professionals

	Proportion of nursing homes with regular contact^**a**^	Number of different professionals in nursing homes^**a**^ Mean (SD); Median [IQR]
**Medical specialists**		
Psychiatrist/Neurologist	90.4% (*n* = 481)^b^	1.6 (0.8); 1 [1–2] (*n* = 426)^b^
Dentist	84.8% (*n* = 479)^b^	1.5 (0.8); 1 [1–2] (*n* = 391)^b^
Urologist	57.3% (*n* = 473)^b^	1.5 (1.0); 1 [1–2] (*n* = 264)^b^
Otorhinolaryngologist	38.8% (*n* = 477)^b^	1.2 (0.6); 1 [1–1] (*n* = 182)^b^
Ophthalmologist	18.1% (*n* = 469)^b^	1.2 (0.5); 1 [1–1] (*n* = 85)^b^
**Allied health professionals**		
Physiotherapist	97.1% (*n* = 484)^b^	3.8 (2.3); 3 [2–5] (*n* = 442)^b^
Speech therapist	85.0% (*n* = 474)^b^	1.8 (0.9); 2 [1–2] (*n* = 382)^b^
Hearing aid acoustician	44.3% (*n* = 474)^b^	1.3 (0.6); 1 [1–1] (*n* = 202)^b^
Optometrist	32.6% (*n* = 469)^b^	1.2 (0.5); 1 [1–1] (*n* = 147)^b^

*SD* standard deviation, *IQR* interquartile range

^a^Regular is defined as at least one contact to one nursing home resident in the last twelve months

^b^Numbers differ because of missing values

### Estimated need for and actual utilization of allied health professionals

**Fig. 3:**
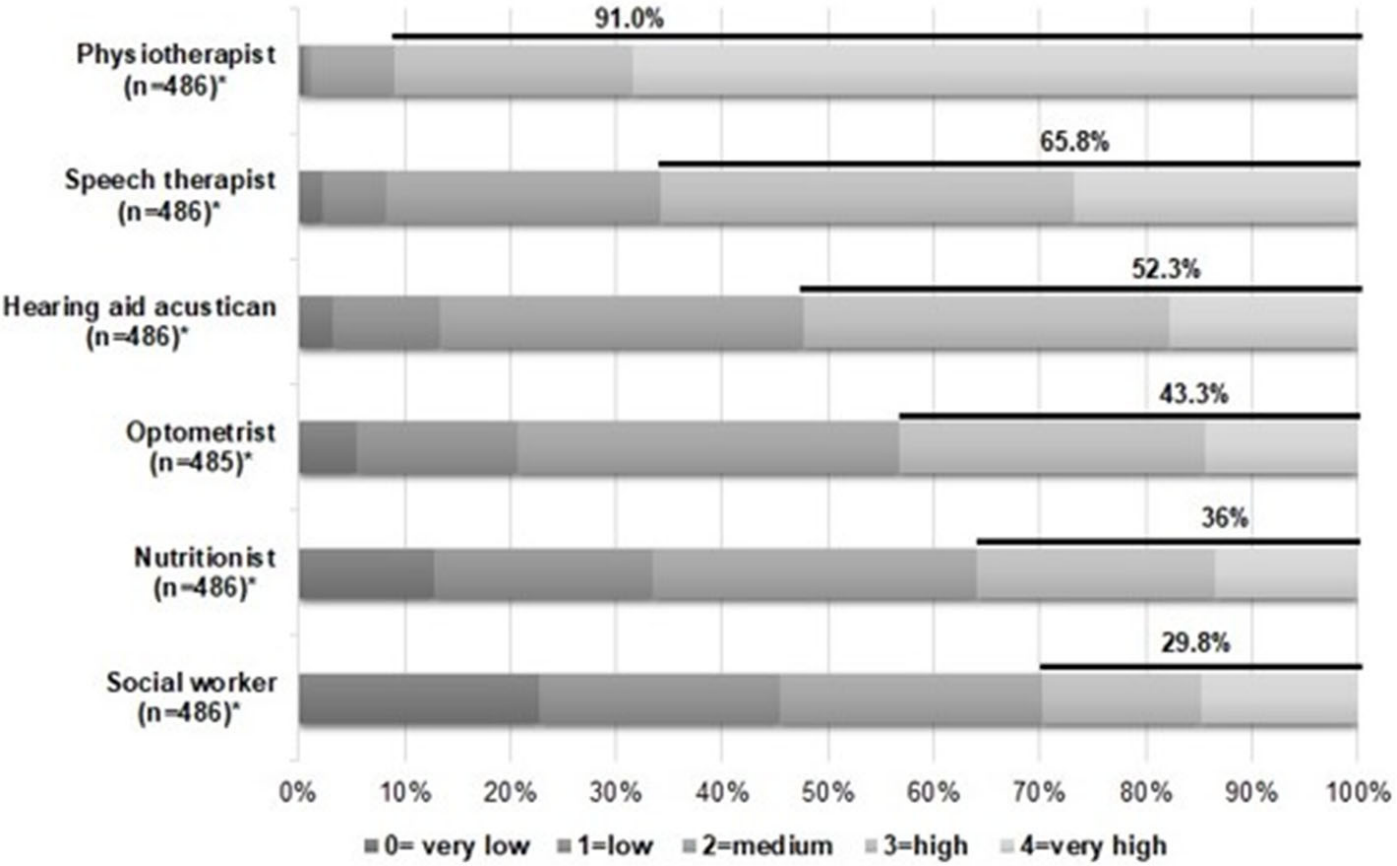
Estimated need for various allied health professionals Detailed legend: *Numbers differ because of missing values

Regarding allied health professionals (Fig. 3; for detailed data see Additional file 3), 91.0% of the respondents estimated a high need for physiotherapists and 65.8% for speech therapists. About half of the participants assessed the need for hearing aid acousticians as high. Between 29.8 and 43.3% of the respondents estimated a high need for social workers, nutritionists and optometrists.

Regarding contacts to allied health professionals (Table 2), almost all nursing homes reported regular contact of residents to a physiotherapist (97.1%). The proportion of nursing homes with regular contact to speech therapists was 85.0%, whereas regular contacts to hearing aid acousticians (44.3%) and optometrists (32.6%) were less common. A mean of 3.8 (SD: 2.3; IQR: 2–5) physiotherapists per nursing home was estimated. The average numbers of speech therapists, optometrists and hearing aid acousticians per nursing home were lower.

## Discussion

In this cross-sectional study, data from 486 nursing homes on the needs and utilization of medical care were assessed. We found that on average nursing homes are served by about 9 different GPs with a large variance among the different facilities, whereas this number is much lower for medical specialists. There were high needs for psychiatrists/neurologists, dentists and physiotherapists, which were also often utilized. High needs for regular visits of urologists and ophthalmologists seem not to be satisfied. Only about half of the respondents saw a high need for speech therapists and hearing aid acousticians.

Regarding medical specialists, 18.1% of the nursing homes reported contacts to an ophthalmologist in the last 12 months. Other German studies found proportions of 6.4 to 19.3% [29, 30] and support our finding. On the other hand, 51.5% of our respondents estimated the need for ophthalmologists in nursing homes as high or very high, suggesting that there is a significant supply gap. This was also supported by other studies and might affect quality of life of nursing homes residents [14, 31]. Larsen et al. found that residents with moderate or severe visual impairment or blindness are significantly more in need of care and have lower mobility than nursing home residents without visual impairment [15]. Visual impairment is also associated with an increased risk of falls, higher rates of depression symptoms and cognitive dysfunction [32–34]. Some eye disorders like cataract or glaucoma are also highly prevalent, and some of these diseases can be treated with simple and effective methods like glasses or medication [14]. The reasons for this supply gap are not clear, but lack of mobile diagnostic equipment might play a role. Collaboration with and visits by optometrists might also be a solution.

In our study, the vast majority of nursing homes (84.8%) have access to dentists, but we did not assess which proportion of residents had contacts. Other German studies reported contact rates of only between 39.6 and 45.5% [22, 29], suggesting that our results might overestimate the actual utilization. Nevertheless, participants see a high need for dentists (73.3%), which is consistent with the results of other studies [9]. Dental problems have a substantial impact on daily activities like eating or speaking. This relationship was shown by Porter et al., who found that nearly 34.4% of nursing home residents suffered from loose or ill-fitting dentures, 40% of dry mouth problems and 17.1% of nursing home residents with remaining teeth reported toothache in the last 6 months [10]. This also highlights the need for nursing staff or GPs performing a regular assessment of dental status, which does not require devices.

Few German studies are available on the contacts of nursing home residents with allied health professionals, and, as far as we know, there is no other study on needs regarding allied health professionals. Physiotherapists and speech therapists are present in almost all nursing homes, a result that is comparable to international studies. In the Netherlands, for instance, physiotherapists and speech therapists are present in 98.9 and 92.0% of nursing homes, respectively [35]. The use of physiotherapy in nursing homes in the USA and the UK is quite a bit lower at 44.0 and 76%, respectively [36, 37]. Interestingly, more than half of the nursing homes in our study do not have a hearing aid acoustician or an optometrist, and the need for both professionals was also estimated to be low. This might suggest that the impact of visual or hearing problems in nursing home residents or the competencies of hearing aid acousticians and optometrists are underestimated by nursing home staff.

GPs play an important role in the medical care for nursing home residents as nearly all residents are regularly seen by GPs [20, 22]. Our results suggest that nursing homes have contacts to many different GPs, which might prevent more regular visits because each GP cares for too few residents per nursing home. However, regular visits and better interdisciplinary communication are supposed to be key factors in improving the medical care for nursing home residents [38]. Interestingly, about 70% of respondents saw the GP as a coordinator of medical care in nursing homes and wanted more regular visits, but only 46.0% agreed that specialist contacts should occur solely after referral by GPs. Moreover only 51.9% of the respondents agreed that medical care by one medical practice leads to a higher quality of care. These results are somewhat inconsistent because on the one hand, nursing homes emphasize the key role of GPs at least to some extent. On the other hand, they want to decide themselves when to contact medical specialists. These findings highlight the fact that a significant proportion of nursing staff seem to not support the idea of a nursing home physician.

In contrast to Germany, in the neighboring Netherlands nursing homes employ their own physicians [39]. Here, nursing home medicine is a separate medical specialty generating skills regarding chronic diseases, geriatric and end-of-life care [40]. This approach is beneficial for the communication between nurses and physicians and allows identifying avoidable life-threatening conditions in order to avoid hospital admissions [41]. Furthermore eye care in Dutch nursing homes is performed not only by ophthalmologists, but also by nursing home physicians, who frequently perform many diagnostic activities, including visual field, near vision and distance vision testing [42]. However, the right of German patients to choose their own physicians might interfere with the implementation of nursing home physicians in Germany.

Interprofessional collaboration between nursing staff, GPs, medical specialists and allied health professionals should be improved in Germany, because good communication leads to better feedback regarding the needs and outcomes of medical care in nursing home residents. One approach for achieving better communication could be more regular requests of therapy reports as part of referrals to allied health professionals. Another approach could be to reduce the number of responsible GPs per nursing home to some extent, which would also mean that these fewer GPs conduct more visits. Also, medical specialists should recognize that visiting nursing home residents is as important as providing medical care to patients visiting their practices.

### Strengths and limitations

The high response is a strength of our study. Moreover, this survey is one of the first studies providing information regarding the needs for medical specialists and allied health professionals. Furthermore, this study is a comprehensive investigation on a national level, including all 16 federal states of Germany.

Our results are based on estimations of nursing staff, and there might be more valid methods for measuring the need for and utilization of medical specialists and allied health professionals. As we did not provide a specific definition of the term “need”, need might be defined differently among the respondents. Moreover, some responses like contacts to dentists may be socially desirable. However, questionnaires were evaluated completely anonymous to reduce social desirability. In contrast to other studies, we did not examine contact frequency per nursing home resident, but contact to at least one resident as well as the number of GPs, medical specialists and health professionals visiting nursing homes within the last twelve months.

## Conclusion

This study shows that there is a high need for medical specialists like dentists, ophthalmologists, urologists and psychotherapists as well as for allied health professionals like physiotherapists and speech therapists in nursing homes. In contrast to psychiatrists and otorhinolaryngologists, where utilization matches estimated needs, needs outweigh utilization for ophthalmologists and urologists. In addition, the needs for speech therapists and dentists are estimated lower in relation to their use. The reasons for the low number of contacts to nursing home residents, especially of ophthalmologists and urologists, are unclear and should be investigated, in particular from the point of view of GPs, medical specialists and allied health professionals and by using qualitative methods.

## Supplementary information

**Additional file 1.** Perceptions on medical care for nursing home residents (detailed data for Fig. 1)

**Additional file 2.** Estimated need for various medical specialists (detailed information for Fig. 2)

**Additional file 3.** Estimated need for various allied health professionals (detailed data for Fig. 3).

## Data Availability

The datasets supporting the conclusions of this article are available from the corresponding author on reasonable request.

## Abbreviations

GP: General Practitioner
IQR: Interquartile range
SD: Standard deviation
