# Supplementary figures and images for: Needs and availability of medical specialists’ and allied health professionals’ visits in German nursing homes: a cross-sectional study of nursing home staff
Source: BMC Health Serv Res. 2020 Apr 21;20:332. doi: 10.1186/s12913-020-05169-7 (PMC7171863; doi:10.1186/s12913-020-05169-7)

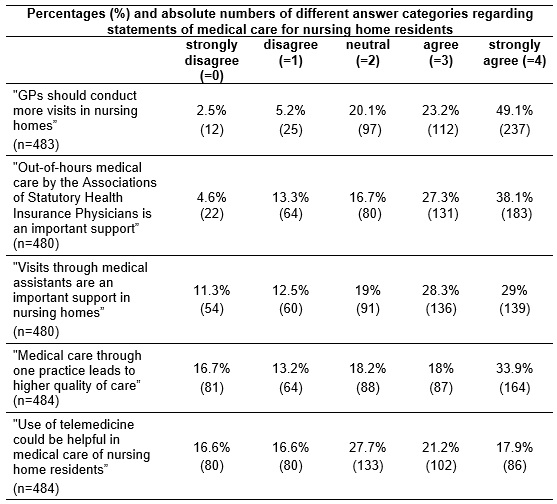

Supplement: Supplementary file 1 — Additional file 1. Perceptions on medical care for nursing home residents (detailed data for Fig. 1) [file 12913_2020_5169_MOESM1_ESM.docx]

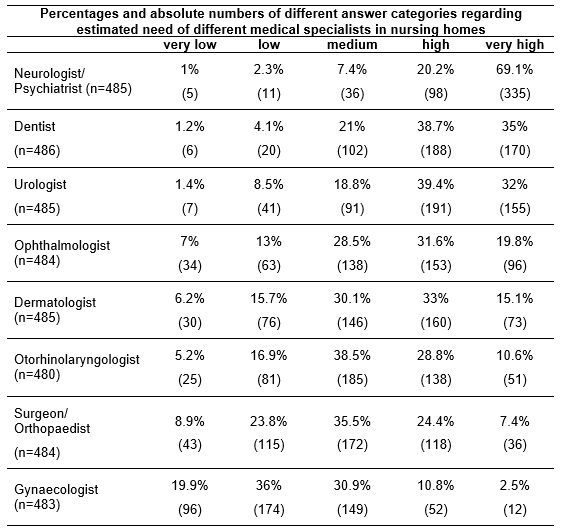

Supplement: Supplementary file 2 — Additional file 2. Estimated need for various medical specialists (detailed information for Fig. 2) [file 12913_2020_5169_MOESM2_ESM.docx]

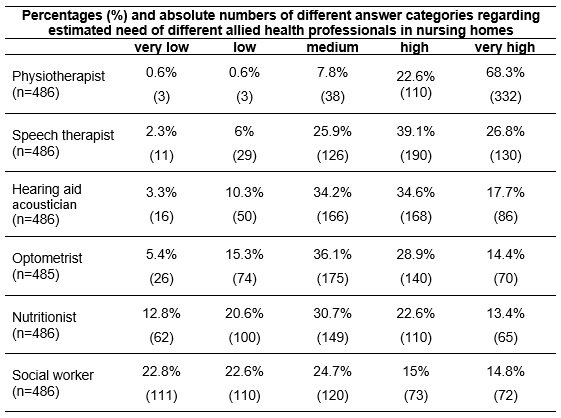

Supplement: Supplementary file 3 — Additional file 3. Estimated need for various allied health professionals (detailed data for Fig. 3). [file 12913_2020_5169_MOESM3_ESM.docx]
